# Time Series Analysis of SARS-CoV-2 Genomes and Correlations among Highly Prevalent Mutations

**DOI:** 10.1128/spectrum.01219-22

**Published:** 2022-09-07

**Authors:** Neha Periwal, Shravan B. Rathod, Sankritya Sarma, Gundeep S. Johar, Avantika Jain, Ravi P. Barnwal, Kinsukh R. Srivastava, Baljeet Kaur, Pooja Arora, Vikas Sood

**Affiliations:** a Department of Biochemistry, SCLS, Jamia Hamdard, New Delhi, India; b Department of Chemistry, Smt. S. M. Panchal Science College, Talod, Gujarat, India; c Department of Zoology, Hansraj College, University of Delhi, New Delhi, India; d Humber College, Toronto, Ontario, Canada; e Delhi Institute of Pharmaceutical Sciences and Research, New Delhi, Delhi, India; f Department of Biophysics, Panjab University, Chandigarh, India; g Division of Medicinal and Process Chemistry, CDRI, Lucknow, Uttar Pradesh, India; h Department of Computer Science, Hansraj College, University of Delhi, New Delhi, India; Karolinska Institutet

**Keywords:** COVID-19, hierarchical clustering, mutations, Pearson correlation, protein dynamics, SARS-CoV-2

## Abstract

The efforts of the scientific community to tame the recent pandemic caused by severe acute respiratory syndrome coronavirus 2 (SARS-CoV-2) seem to have been diluted by the emergence of new viral strains. Therefore, it is imperative to understand the effect of mutations on viral evolution. We performed a time series analysis on 59,541 SARS-CoV-2 genomic sequences from around the world to gain insights into the kinetics of the mutations arising in the viral genomes. These 59,541 genomes were grouped according to month (January 2020 to March 2021) based on the collection date. Meta-analysis of these data led us to identify significant mutations in viral genomes. Pearson correlation of these mutations led us to the identification of 16 comutations. Among these comutations, some of the individual mutations have been shown to contribute to viral replication and fitness, suggesting a possible role of other unexplored mutations in viral evolution. We observed that the mutations 241C>T in the 5′ untranslated region (UTR), 3037C>T in nsp3, 14408C>T in the RNA-dependent RNA polymerase (RdRp), and 23403A>G in spike are correlated with each other and were grouped in a single cluster by hierarchical clustering. These mutations have replaced the wild-type nucleotides in SARS-CoV-2 sequences. Additionally, we employed a suite of computational tools to investigate the effects of T85I (1059C>T), P323L (14408C>T), and Q57H (25563G>T) mutations in nsp2, RdRp, and the ORF3a protein of SARS-CoV-2, respectively. We observed that the mutations T85I and Q57H tend to be deleterious and destabilize the respective wild-type protein, whereas P323L in RdRp tends to be neutral and has a stabilizing effect.

**IMPORTANCE** We performed a meta-analysis on SARS-CoV-2 genomes categorized by collection month and identified several significant mutations. Pearson correlation analysis of these significant mutations identified 16 comutations having absolute correlation coefficients of >0.4 and a frequency of >30% in the genomes used in this study. The correlation results were further validated by another statistical tool called hierarchical clustering, where mutations were grouped in clusters on the basis of their similarity. We identified several positive and negative correlations among comutations in SARS-CoV-2 isolates from around the world which might contribute to viral pathogenesis. The negative correlations among some of the mutations in SARS-CoV-2 identified in this study warrant further investigations. Further analysis of mutations such as T85I in nsp2 and Q57H in ORF3a protein revealed that these mutations tend to destabilize the protein relative to the wild type, whereas P323L in RdRp is neutral and has a stabilizing effect. Thus, we have identified several comutations which can be further characterized to gain insights into SARS-CoV-2 evolution.

## INTRODUCTION

A novel coronavirus first appeared in Wuhan, China, in December 2019 and became a public health emergency of international concern. Since its emergence, the virus has caused catastrophe across the globe. This virus, known as severe acute respiratory syndrome coronavirus 2 (SARS-CoV-2), has infected nearly 486 million people and killed more than 6.3 million globally [WHO Coronavirus (COVID-19) Dashboard] as of 13 July 2022. Of the seven known coronaviruses—human coronavirus OC43 (HCoV-OC43), human coronavirus-229E (HCoV-229E), human coronavirus-HKU1 (HCoV-HKU1), human coronavirus-NL63 (HCoV-NL63), severe acute respiratory syndrome coronavirus (SARS-CoV), middle east respiratory syndrome coronavirus (MERS-CoV), and severe acute respiratory syndrome coronavirus 2 (SARS-CoV-2), (1)—SARS-CoV-2 is highly pathogenic to humans (2). This virus has linear, positive-sense, single-strand RNA (ssRNA) as its genetic material, which is 29,903 bp long and is encapsulated by the nucleocapsid protein, which is one of the four structural proteins, the others being spike, envelope, and membrane proteins (3). Once the virus gains entry into the cell, two viral polyproteins, open reading frame 1a (ORF1a) and ORF1ab proteins, are formed. These polyproteins are then cleaved by the viral proteases into 16 nonstructural proteins, which initiate the process of viral replication and transcription. Apart from the viral nonstructural proteins, SARS-CoV-2 encodes 11 accessory proteins that play a key role in the viral pathogenesis (4).

Among the nonstructural proteins of SARS-CoV, nsp14, along with nsp10 and nsp12, plays a key role in maintaining the integrity of the viral RNA, resulting in fewer mutations than in other RNA viruses (5, 6). Despite the fact that SARS-CoV-2 mutates at a slower pace, this virus has evolved into numerous variants since the onset of the pandemic (7). The continuous evolution of SARS-CoV-2 has hindered the efforts of the scientific community to design vaccines and effective antivirals against it (8). Since mutations are one of the key factors driving the virus’ evolution, understanding the kinetics of the mutations is imperative. Several studies have identified a large number of genetic variations, including missense mutations, synonymous mutations, insertions, and deletions, in the genomic sequences of SARS-CoV-2. The most common types of variations along the viral genome are reported to be missense and synonymous mutations (9). Although synonymous mutations may not have a direct impact on protein function, they have the potential to alter codon usage and translational frequency, as well as being able to affect the binding kinetics of microRNAs. Furthermore, it was speculated that the mutations in the 5′ untranslated region (UTR) may alter viral transcription, replication, and folding of the genomic ssRNA sequences (10). Genome analysis of SARS-CoV-2 revealed a substantial mutation bias toward uracil, which might be caused by improved immunogenicity, selection for greater expression, and better mRNA stability (11).

Viral transmission rates are rapidly increasing as the virus evolves. For instance, a single mutation (D614G) in the spike protein has been shown to increase the infectivity of SARS-CoV-2 (12). The appearance of multiple mutations in the same haplotype might lead to possible correlations among these mutations. It has been shown that comutations Y449S and N501Y in the spike protein can lead to reduced infectivity and play a major role in disrupting the antibody-mediated virus neutralization (13). This implies that mutations can have a synergistic effect, resulting in enhanced viral fitness and immune escape. Therefore, understanding the correlations among the mutations in the viral genome might lead to a better understanding of viral pathogenesis and evolution.

Several studies on this topic have been published. Zuckerman et al. analyzed 371 Israeli genomic sequences from February 2020 to April 2020 and observed correlations among identified mutations with that of known clade-defining ones (14). Wang et al. analyzed pairwise comutations in the most frequent 11 missense mutations that were prevalent in the United States (15). The authors included 12,754 SARS-CoV-2 sequences from the United States and identified missense mutations. In another study, Rahman et al. analyzed 324 complete and nearly complete SARS-CoV-2 genomic sequences which were isolated between 30 March 2020 and 7 September 2020 (16). They identified 3037C>T as the most frequent mutation, as it occurred in 98% of isolates. Though synonymous, this mutation was shown to co-occur with 3 other mutations, including 241C>T, 14408C>T, and 23403A>G. In another study, Chen et al. (17) analyzed 261,323 sequences of SARS-CoV-2 from across the globe to study the evolution of the virus. The authors observed that the initial SARS-CoV-2 M genotype ignited the COVID-19 outbreak. The M genotype harbored two concurrent mutations and was transformed to WE1 by acquiring four additional concurrent mutations (17). The WE1 genotype further evolved into WE1.1 by incorporating three additional concurrent mutations.

**FIG 1:**
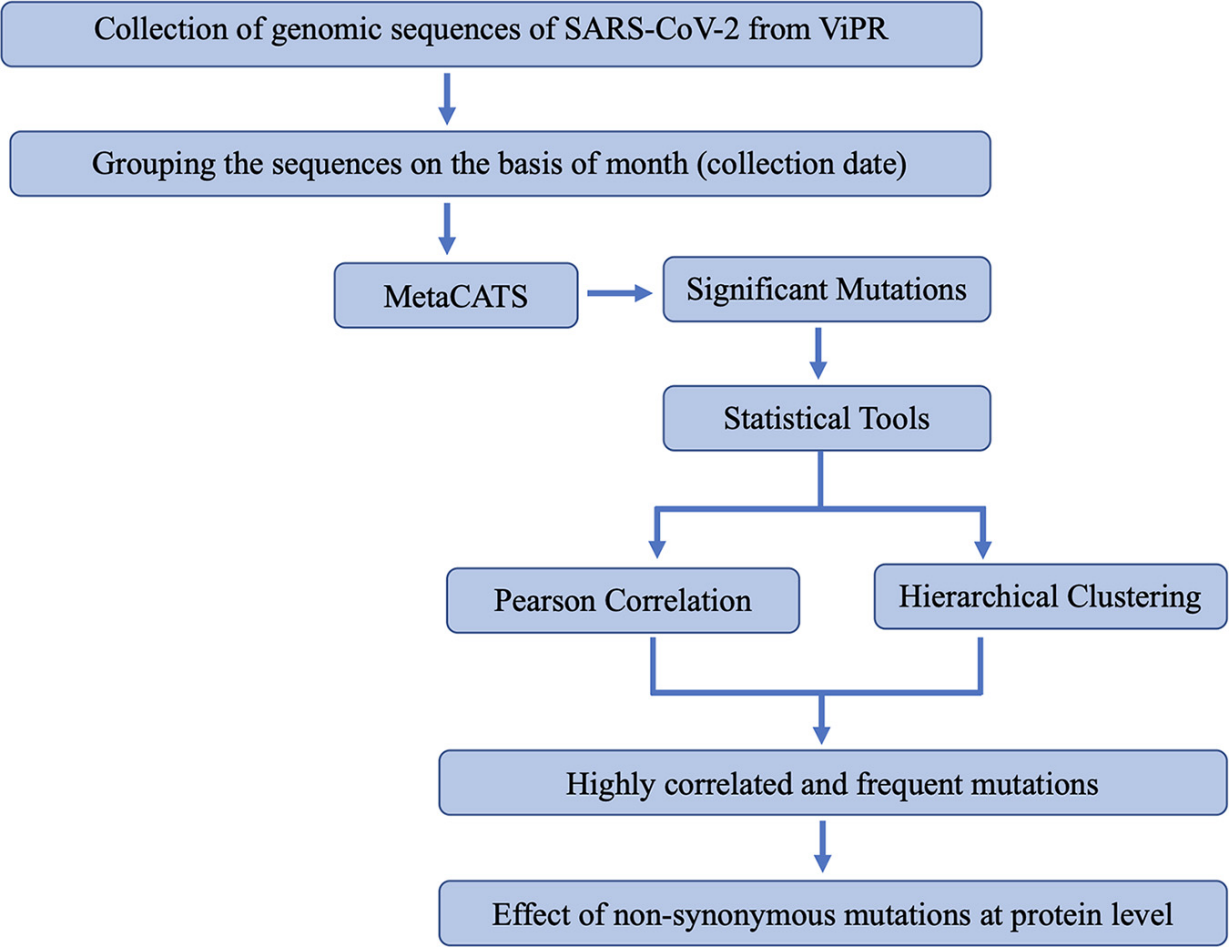
Methodology used in this study. The SARS-CoV-2 complete genome sequences were obtained from the ViPR and grouped by month based on the collection date. The meta-analysis was then performed in a pairwise manner, i.e., comparing January 2020 to each month through March 2021, to identify the highly significant mutations arising among the genomic sequences of SARS-CoV-2. Once the significant mutations were identified, we used Pearson correlation- and hierarchical clustering-based approaches to identify correlations and clusters among the highly significant mutations. The effect of these mutations on the wild-type protein was then studied using several computational tools, including PredictSNP, ENCoM ΔΔ*S*_Vib_, DynaMut, ENCoM ΔΔ*G*, mCSM ΔΔ*G*, DUET ΔΔ*G*, and SAAFEC-SEQ.

Some of the studies mentioned above were performed with SARS-CoV-2 genomic sequences obtained from a specific region, whereas some focused on the few significant missense mutations only. We hypothesized whether a similar trend could be observed with the genomic sequences of SARS-CoV-2 collected from around the world. In order to gain a better understanding of the origin of mutations in SARS-CoV-2 sequences, we analyzed viral genomic sequences in a time series-dependent manner. Meta-analysis of these SARS-CoV-2 genomic sequences led us to the identification of significant mutations. We performed two widely used statistical tools: Pearson correlation, which identified the comutations in the viral genome, and hierarchical clustering, which measured similarities between these mutations and grouped them in clusters. *In silico* protein dynamics was then used for the characterization of the impact of these mutations on their respective proteins (Fig. 1).

## RESULTS AND DISCUSSION

### SARS-CoV-2 genomes.

**FIG 2:**
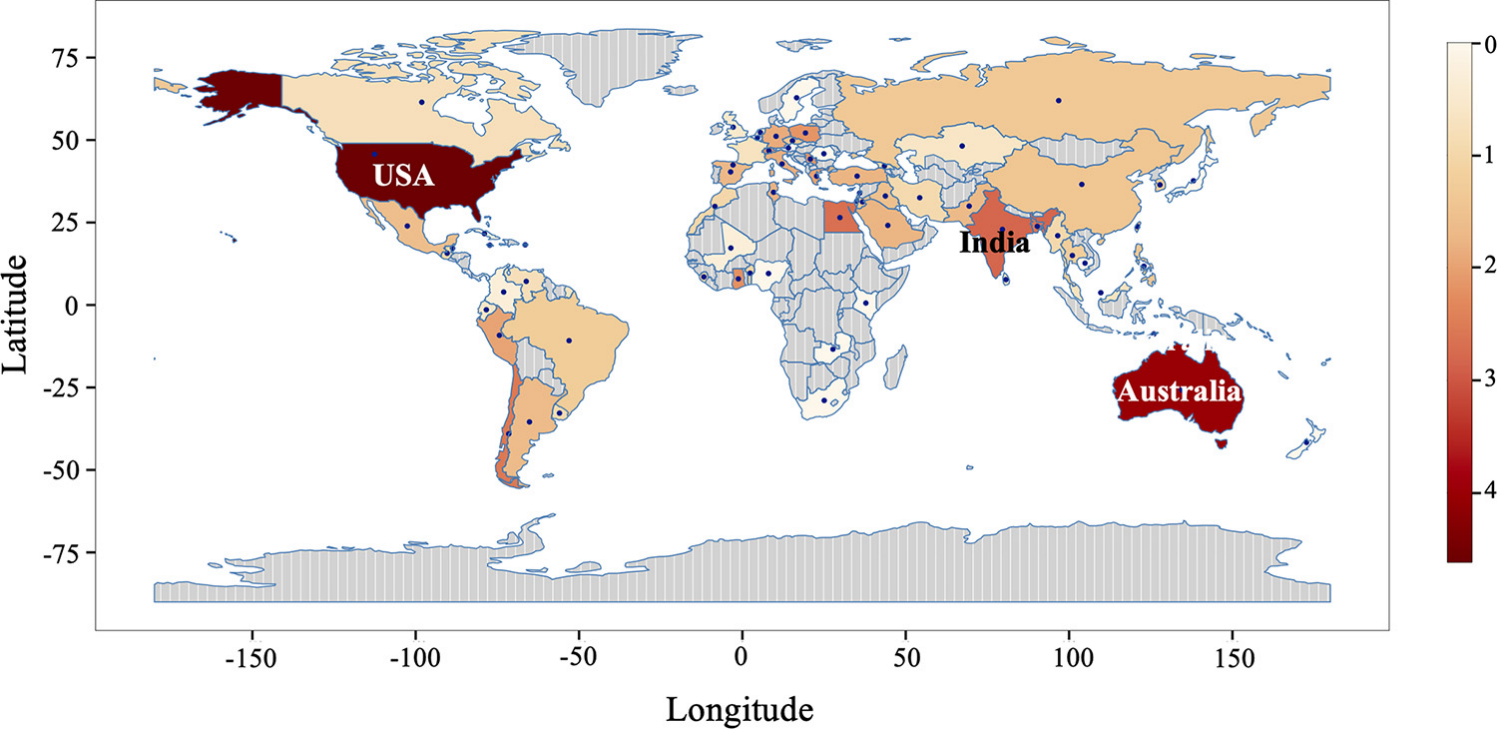
Geographical distribution of the SARS-CoV-2 genomic sequences used in this study. The color bar represents the frequency (log_10_) of sequences. The areas that contributed the maximum number of genomic sequences in this study are represented by dark red shading, whereas areas that contributed fewer genomic sequences are represented by light orange.

In order to understand the kinetics of highly prevalent mutations in the SARS-CoV-2 circulating genomes, we sought to analyze these genomic sequences in a time series manner. There can be a considerable time lapse between sample collection and sample processing; therefore, we used the sample collection month to classify the SARS-CoV-2 genomic sequences. We included a total of 59,541 SARS-CoV-2 genomes that were collected from January 2020 until March 2021 and grouped them by month based on sample collection (see Table S1 in the supplemental material). The number of SARS-CoV-2 genomic sequences from each country was visualized on a world map (Fig. 2). The global distribution of the samples revealed that the majority of the samples were from the United States, followed by Australia, India, and Egypt.

### Identification of significant mutations in the SARS-CoV-2 genomes.

**FIG 3:**
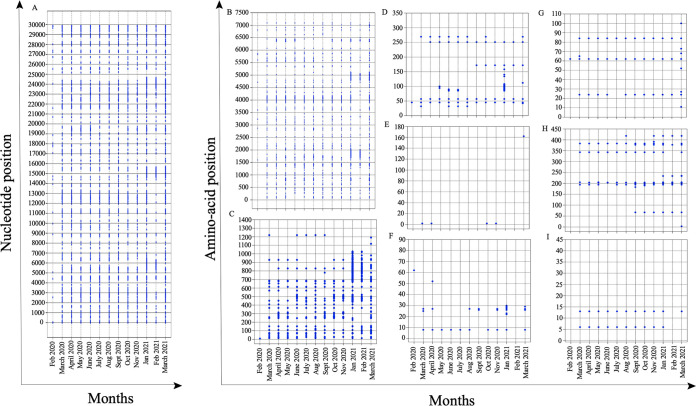
Mutations in the SARS-CoV-2. (A) Mutations at the nucleotide level across the whole genome of SARS-CoV-2. (B to I) Nonsynonymous mutations at the amino acid level for the SARS-CoV-2 proteins (B) ORF1ab, (C) spike protein, (D) ORF3a protein, (E) membrane protein, (F) ORF6 protein, (G) ORF8 protein, (H) nucleocapsid protein, and (I) ORF10 protein. Some mutations appeared early in the pandemic and were consistently present throughout the study.

Once the SARS-CoV-2 genomic sequences were grouped on the basis of the sample collection month, we used the META-CATS algorithm to identify significant mutations among the genomes. This algorithm compares two different data sets to identify significant mutations among them. As the genomic sequences collected at the start of the pandemic tend to be very similar to the parent sequence, all the SARS-CoV-2 genomic sequences collected in January 2020 were grouped together to form a control group. The sequences obtained in subsequent months were then analyzed against the sequences from the control group to identify significant mutations in SARS-CoV-2 genomes of that particular month. We obtained significant mutations for each month except December 2020 (Fig. 3A). Since mutations at the nucleotide level might not lead to changes in amino acids due to the degeneracy of the genetic code, we focused our attention on the mutations at the amino acid level (Fig. 3B to I). We identified 940 unique mutations at the amino acid level which were unevenly distributed among the genome of SARS-CoV-2. Our analysis identified 610, 256, 33, 2, 11, 10, 16, and 2 mutations in the ORF1ab, spike, ORF3a, membrane, ORF6, ORF8, nucleocapsid, and ORF10 proteins of SARS-CoV-2, respectively. As the length of SARS-CoV-2 proteins is highly variable, we calculated the frequency of the mutations at the amino acid level in order to understand their distribution in the viral proteins. We observed that the spike protein had the highest frequency of mutations (20.10%), followed by ORF6 (18%) and ORF1ab (8.59%) (Table 1). We observed that the membrane protein of SARS-CoV-2 had the fewest mutations compared to the other proteins, suggesting that this region might be highly conserved among SARS-CoV-2 variants. The recently emerged SARS-CoV-2 mutant Omicron has been shown to have more than 40 mutations in its spike protein, suggesting that the protein is highly amenable to mutations (18).

**TABLE 1:** Frequency of unique significant mutations in various SARS-CoV-2 proteins

Protein	Length (aa)	No. of unique mutations	Frequency^*a*^
Orf1ab	7,096	610	8.59
S	1,273	256	20.10
Orf3a	275	33	12
M	222	2	0.90
Orf6	61	11	18.0
Orf8	121	10	8.2
N	419	16	3.8
Orf10	38	2	5.1

a Calculated by dividing the number of unique mutations by the length of the respective protein and then multiplying by 100.

### Correlation among significant mutations in SARS-CoV-2 genomes.

Co-occurrence of several mutations has been shown to modulate the function of the proteins (19). Therefore, we sought to understand whether there was any correlation among the significant mutations that we identified in this study. For this purpose, we utilized two well-established statistical approaches: Pearson correlation, which measures the correlation coefficient (positive or negative) between two mutations, and hierarchical clustering, which groups similar mutations into clusters.

### (i) Pearson correlation coefficient.

**FIG 4:**
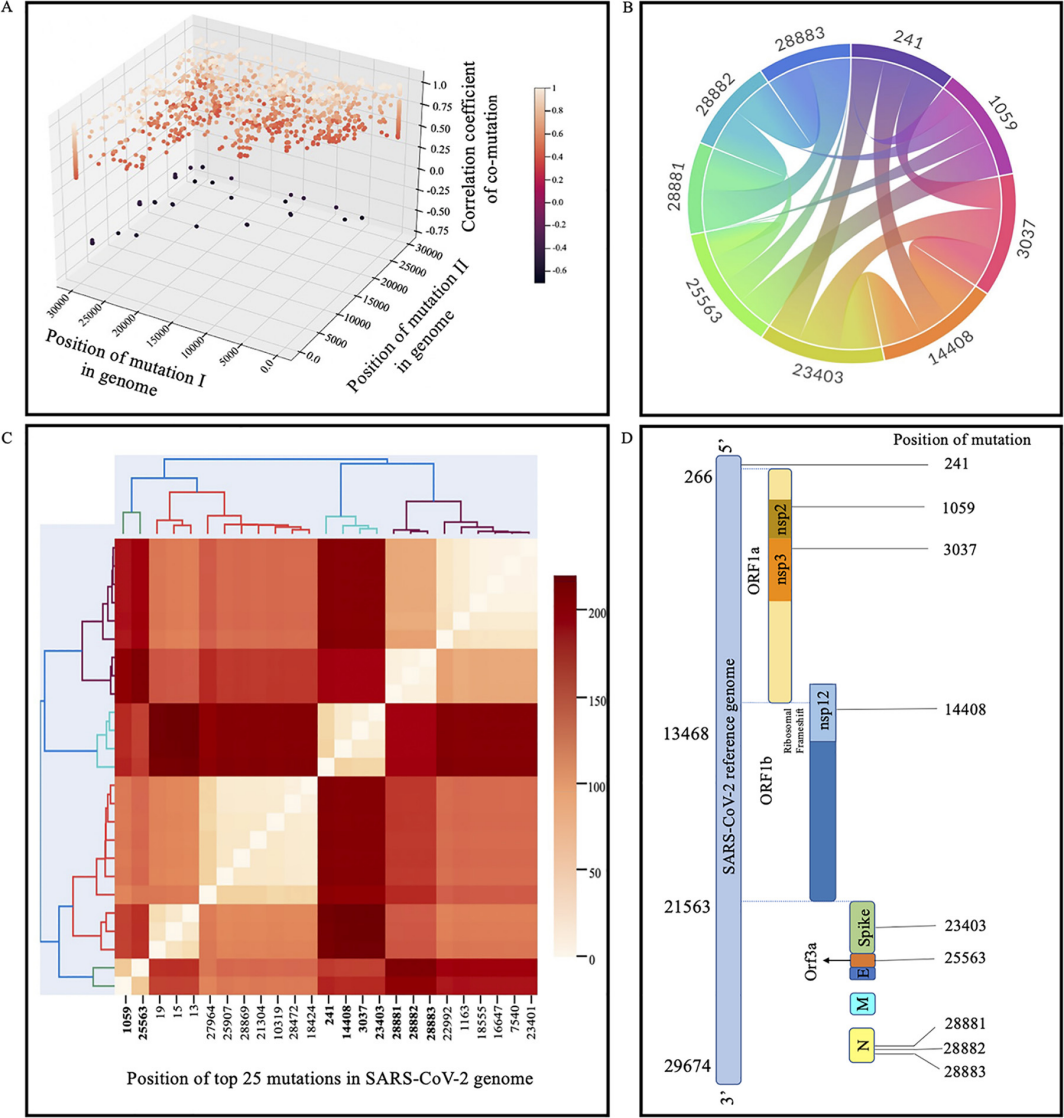
Significant mutations that were correlated and clustered with each other by using statistical approaches (Pearson correlation and hierarchical clustering). (A) Three-dimensional plot showing the comutations having Pearson correlation coefficients with absolute values of >0.4. The degree of correlation is reflected by the color. The correlation coefficient ranges from −1 to +1; absolute values closer to 1 have a higher correlation than those closer to 0. (B) Chord plot representing comutations with absolute correlation coefficients of >0.4 and occurring in more than 30% of the genomes used in the study. (C) Hierarchical clustering of the 25 most significant mutations obtained from ViPR having a frequency of >10%. The *x* axis represents the significant mutations. The colors represent the distances among the mutations. The comutations identified in this study are highlighted in bold. (D) Cartoon showing the positions of mutations in the viral genome.

Analysis of SARS-CoV-2 sequences in a time series manner led us to the identification of several significant mutations. In order to identify the correlation among these mutations, Pearson correlation was performed on a binary matrix, with 1 representing significant mutations and 0 representing no mutations in SARS-CoV-2 genomes. The correlation value ranges from −1.0 to +1.0, with negative values indicating negative correlation and positive values indicating positive correlation. Additionally, absolute values closer to 1 indicate a very strong correlation. The results obtained from the Pearson correlation were then filtered to obtain only comutations where the absolute value of the correlation coefficient was greater than 0.4. Using this criterion, we obtained 2,205 comutations (Fig. 4A). It was observed that the frequency of the majority of these comutations was very low. For instance, a comutation at positions 21306 and 22995 with an absolute value of the correlation coefficient of >0.4 but occurrence in less than 5% of the genomes might not be of interest. Therefore, we considered only comutations that were present in >30% of genomes for further study. Using this stringent criterion, we identified 16 comutations that had an absolute value of the correlation coefficient of >0.4, with each mutation of the comutation being present in >30% of the genomes (Fig. 4B and Table 2). It was further observed that six comutations were present in >89% of the genomes, suggesting their possible role in viral fitness. Our analysis captured a highly prevalent mutation in the spike protein (D614G) that has nearly replaced the wild-type sequence and is known to increase viral infectivity (12). The identification of the D614G mutation further validated our approach and prompted us to further explore other comutations that were identified.

**TABLE 2:** Correlations among various unique mutations in SARS-CoV-2 genomes^*a*^

Position of mutation:		Correlation value of comutation	% of genomes with:		
I	II		Mutation I	Mutation II	Both mutations
241 (5′ UTR)	3037 (nsp3)	0.760	91.054	89.889	87.16
241 (5′ UTR)	23403 (spike)	0.758	91.054	90.082	87.26
241 (5′ UTR)	14408 (RdRp)	0.733	91.054	89.385	86.61
1059 (nsp2)	25563 (ORF3a)	0.863	40.022	46.948	39.33
1059 (nsp2)	28882 (nucleocapsid)	−0.534	40.022	30.234	0.07
1059 (nsp2)	28881 (nucleocapsid)	−0.535	40.022	30.381	0.10
1059 (nsp2)	28883 (nucleocapsid)	−0.535	40.022	30.259	0.06
3037 (nsp3)	23403 (spike)	0.976	89.889	90.082	88.52
3037 (nsp3)	14408 (RdRp)	0.943	89.889	89.385	87.87
14408 (RdRp)	23403 (spike)	0.943	89.385	90.082	87.97
25563 (ORF3a)	28882 (nucleocapsid)	−0.613	46.948	30.234	0.13
25563 (ORF3a)	28881 (nucleocapsid)	−0.614	46.948	30.381	0.17
25563 (ORF3a)	28883 (nucleocapsid)	−0.614	46.948	30.234	0.11
28881 (nucleocapsid)	28882 (nucleocapsid)	0.995	30.381	30.234	29.77
28881 (nucleocapsid)	28883 (nucleocapsid)	0.994	30.381	30.259	29.77
28882 (nucleocapsid)	28883 (nucleocapsid)	0.997	30.234	30.259	29.77

a The comutations shown have a correlation value of <−0.4 and >0.4 and are present in >30% of the genomes. Comutations showing positive correlations are present in the majority of the same genomes. As expected, comutations showing negative correlations are not present in the same genomes.

### (ii) Hierarchical clustering.

In order to garner confidence and validate our results that were obtained using Pearson correlation, we used another statistical tool to group the significant mutations in clusters. Since hierarchical clustering is a computationally intensive process, we analyzed only the 25 most significant mutations that were present in >10% of the genomes used in this study (Table 3). Similar to the results obtained from Pearson correlation, hierarchical clustering analysis led to the grouping of the mutations in clusters that possess similarities. Here, we analyzed only clusters in which the frequency of each mutation was greater than 30% of genomes (Fig. 4C). The mutations at positions 241, 14408, 3037, and 23403 in the SARS-CoV-2 genome form a cluster and are the most common concurrent mutations. Since both the statistical tools provided similar results, we then focused our attention on these mutations to acquire an in-depth understanding of them. The positions of the nine mutations in the SARS-CoV-2 genomes are depicted in Fig. 4D.

**TABLE 3:** The 25 most significant mutations and their positions in SARS-CoV-2 genomes

No.	Position	Gene	No. of sequences^*a*^
1	241	5′ UTR	50,771
2	23403	Spike	50,229
3	3037	ORF1ab	50,121
4	14408	ORF1ab	49,840
5	25563	ORF3a	26,178
6	1059	ORF1ab	22,316
7	28881	Nucleocapsid	16,940
8	28883	Nucleocapsid	16,872
9	28882	Nucleocapsid	16,858
10	27964	ORF1ab	10,985
11	1163	ORF1ab	9,098
12	10319	ORF1ab	8,882
13	18555	ORF1ab	8,772
14	28869	Nucleocapsid	8,770
15	16647	ORF1ab	8,755
16	23401	Spike	8,746
17	7540	ORF1ab	8,729
18	18424	ORF1ab	8,758
19	28472	Nucleocapsid	8,572
20	21304	ORF1ab	8,320
21	25907	ORF3a	8,236
22	22992	Spike	8,146
23	19	5′ UTR	6,637
24	15	5′ UTR	5,662
25	13	5′ UTR	5,069

a Number of genomic sequences in which the mutation in present.

### Frequency and global distribution of highly correlated and frequent significant mutations.

**FIG 5:**
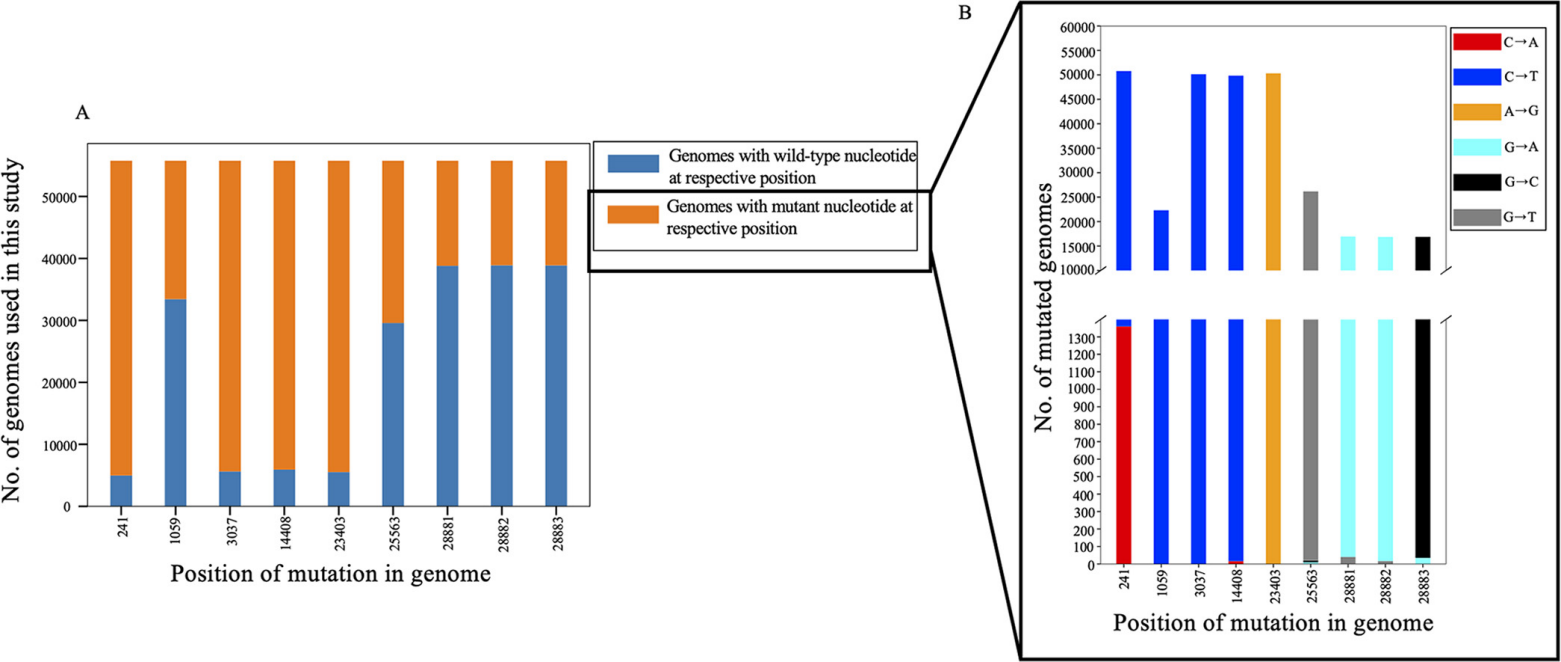
Stacked bar chart showing the distribution of genomes with a given mutation. (A) The total length of the bar represents the total number of SARS-CoV-2 genomic sequences. The length of the blue bar represents the number of genome sequences with wild-type nucleotides at that position; the length of the orange bar represents the number of genome sequences with a given mutation (mutated nucleotide). (B) Bar length represents the number of genomic sequences that have a mutation at a particular position. A position with a single bar indicates that the wild-type nucleotide is substituted by a single nucleotide. A position with multiple bars indicates that the wild-type nucleotide is substituted by more than one nucleotide. The lengths of the different-color bars represent the degree of substitution.

**FIG 6:**
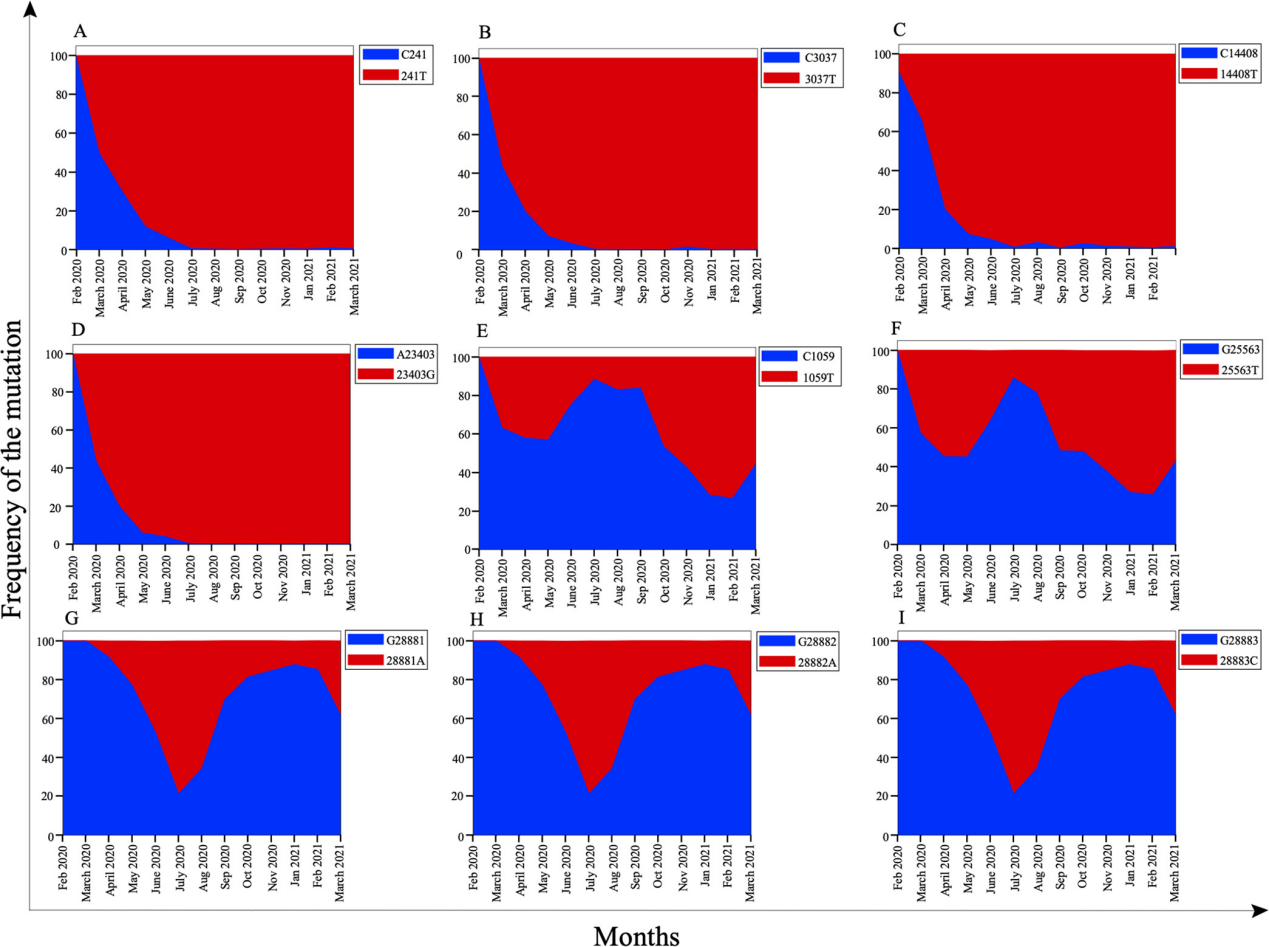
Running monthly counts of the sampled sequences exhibiting significant mutations (that have a correlation coefficient with an absolute value of >0.4 and are present in >30% of the SARS-CoV-2 genomes under study) between 1 February 2020 and 31 March 2021, as follows: (A) 241C>T in the 5′ UTR; (B) 3037C>T in nsp3 (ORF1ab protein); (C) 14408C>T in RdRp (ORF1ab protein); (D) 23403A>G in the spike protein; (E) 1059C>T in nsp2 (ORF1ab protein); (F) 25563G>T in the ORF3a protein; (G) 28881G>A in the nucleocapsid protein; (H) 28882G>A in the nucleocapsid protein; and (I) 28883G>C in the nucleocapsid protein. Blue represents the SARS-CoV-2 genomic sequences having wild-type nucleotides at a particular position, whereas red represents the sequences having mutant nucleotides at a particular position.

Once the comutations that have a correlation coefficient with an absolute value of >0.4 and are present in >30% of genomes were identified, we sought to investigate the frequency of each mutation in SARS-CoV-2 genomes. There are 9 mutations that constitute the 16 comutations. The nucleotide positions where these mutations occur are 241, 1059, 3037, 14408, 23403, 25563, 28881, 28882, and 28883. Analysis of each position in the genome revealed that mutations at 241, 3037, 14408, and 23403 almost completely replaced the wild-type sequences (Fig. 5A). Analysis of the genome revealed that the major nucleotide change that occurred in around 97% of the mutated population at position 241 in the SARS-CoV-2 genome was 241C>T (Fig. 5B). However, in the remaining 3% of the mutated SARS-CoV-2 sequences, 241C>A was observed (Fig. 5 and Table 4). Since the frequency of the major mutations was much higher than that of the minor mutation at the same nucleotide position, we considered the major mutation for further study. We investigated the global distribution of all nine mutations among the circulating SARS-CoV 2 genomes and found that the mutation 241T in the 5′ UTR completely replaced the wild-type nucleotide, C241, as early as June-July 2020 (Fig. 6A). Similar trends were observed with the mutations 3037C>T, 14408C>T, and 23403A>G in nsp3, RNA-dependent RNA polymerase (RdRp), and spike proteins, respectively (Fig. 6B to D). The prevalence of these mutations in the SARS-CoV 2 circulating genomes suggests their critical role in viral pathogenesis. Other mutations, including one in nsp2 (1059C>T), one in ORF3a (25563C>T), and three in nucleocapsid protein (28881G>A, 28882G>A, and 28883G>C) showed a mosaic pattern of global distribution that increased over time (Fig. 6E to I).

**TABLE 4:** Frequency of major and minor nucleotide substitutions for a specific mutation

Position of mutation in the genome	Genomic region	Nucleotide before mutation (reference)	Nucleotide(s) after mutation		Amino acid(s) in:		
					Wild-type protein (reference)	Mutant protein	
			Major	Minor		Major	Minor
241	5′ UTR	C241	241T	241A			
1059	nsp2 (ORF1ab)	C1059	1059T		T265	265I	
3037	nsp3 (ORF1ab)	C3037	3037T		F106	106F	
14408	nsp12 (ORF1ab)	C14408	14408T	14408A	P323	323L	323H
23403	Spike	A23403	23403G		D614	614G	
25563	ORF3a	G25563	25563T	25563A/25563C	Q57	57H	57Q/57H
28881	Nucleocapsid	G28881	28881A	28881T	R203	203K	203M
28882	Nucleocapsid	G28882	28882A	28882T	R203	203R	203S
28883	Nucleocapsid	G28883	28883C	28883A	G204	204R	204R

### Mutation in the 5′ UTR.

The untranslated region of the viral genome plays a vital role in viral replication. This region has been shown to form various secondary structures to allow the binding of cellular and viral proteins, thereby regulating the translation of viral proteins (20, 21). Therefore, any mutation in these highly conserved regions has the potential to regulate viral replication. Statistical approaches revealed that the mutation 241C>T was closely correlated with three different mutations, 3037C>T, 14408C>T, and 23403A>G, in the nsp2, RdRp, and spike genes, respectively, of SARS-CoV-2. Remarkably, it can be observed that the correlation coefficient of mutation 241C>T with all the other mutations mentioned above was >0.75, pointing toward a very strong correlation. Additionally, these mutations were found in >89% of the genomes, further suggesting their critical role in viral evolution. These observations were further supported by hierarchical clustering, in which these mutations were clustered together. Our results are in agreement with published studies that have shown similar correlations among these mutations (14). However, these studies were conducted on the genomes of viruses from various countries, including Israel, the United States, and Bangladesh, whereas our analysis was carried out with SARS-CoV-2 genomes obtained globally. The correlation of the 241C>T mutation with frequently occurring mutations in the SARS-CoV-2 genomes points to its role in viral pathogenesis and fitness.

### Mutation in nsp2.

The protein nsp2 of SARS-CoV-2 was recently shown to be associated with host proteins involved in vesicle trafficking. It was also proposed that targeting the interactions of viral nucleocapsid proteins nsp2 and nsp8 with the host translational machinery might have therapeutic effects (22). Therefore, understanding the dynamics of nsp2 is essential. Our analysis revealed that the mutation 1059C>T in nsp2 was both positively and negatively correlated with other mutations in SARS-CoV-2. As described in Table 2, the mutation 1059C>T (T85I) in the nsp2 gene was positively correlated with 25563G>T (Q57H) in the ORF3a gene with a correlation coefficient of 0.863 and was present in >39% of the genomes, suggesting that the co-occurrence of these mutations might play a role in viral evolution. These observations are in agreement with earlier studies where co-occurrence of 1059C>T with 25563G>T was observed in nearly 70% of COVID-19 cases across the United States (15). Additionally, we observed that the 1059C>T (T85I) mutation in the nsp2 gene was negatively correlated with three mutations—28881G>A (R203K), 28882G>A (R203R), and 28883G>C (G204R)—in the nucleocapsid gene. Though the co-occurrence of these mutations was also established in another study (14), in this study, we show that these mutations are negatively correlated. The negative correlation among these mutations suggests that in a single haplotype, only one of them can occur. The analysis of data revealed the co-occurrence of 1059C>T with 28881G>A, 28882G>A, and 28883G>C in 0.10, 0.07 and 0.06% of the genomes, respectively. Therefore, it would be interesting to further investigate the negative relationship among these mutations under experimental conditions.

**FIG 7:**
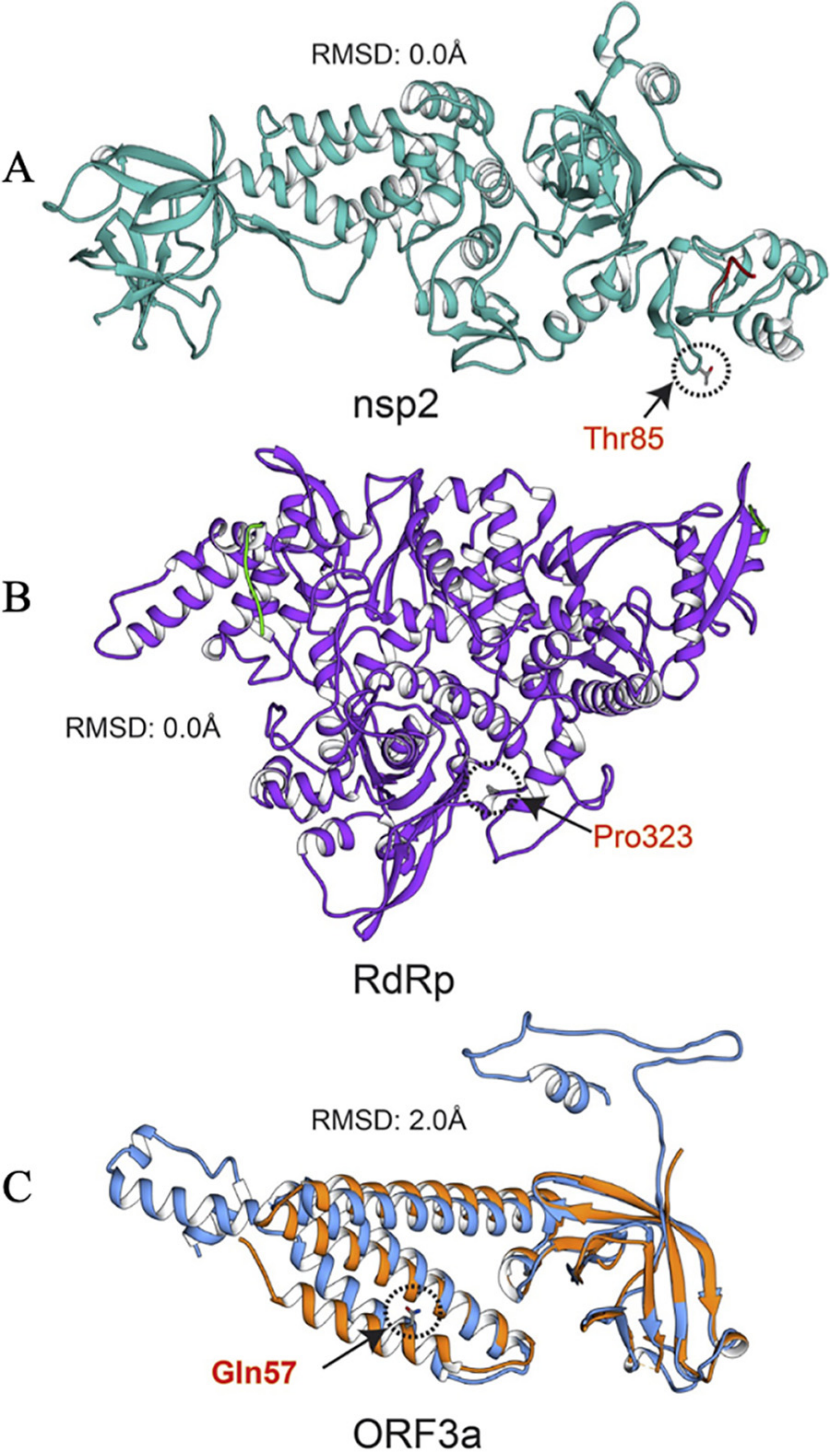
Structure alignments of crystal structure and the modeled proteins. (A) nsp2 crystal and modeled structures are in cyan and red, respectively. (B) RdRp crystal and modeled structures are in purple and red, respectively. (C) ORF3a crystal and modeled structures are in orange and blue, respectively. Mutation positions are circled, and mutant residues are represented in stick form.

**FIG 8:**
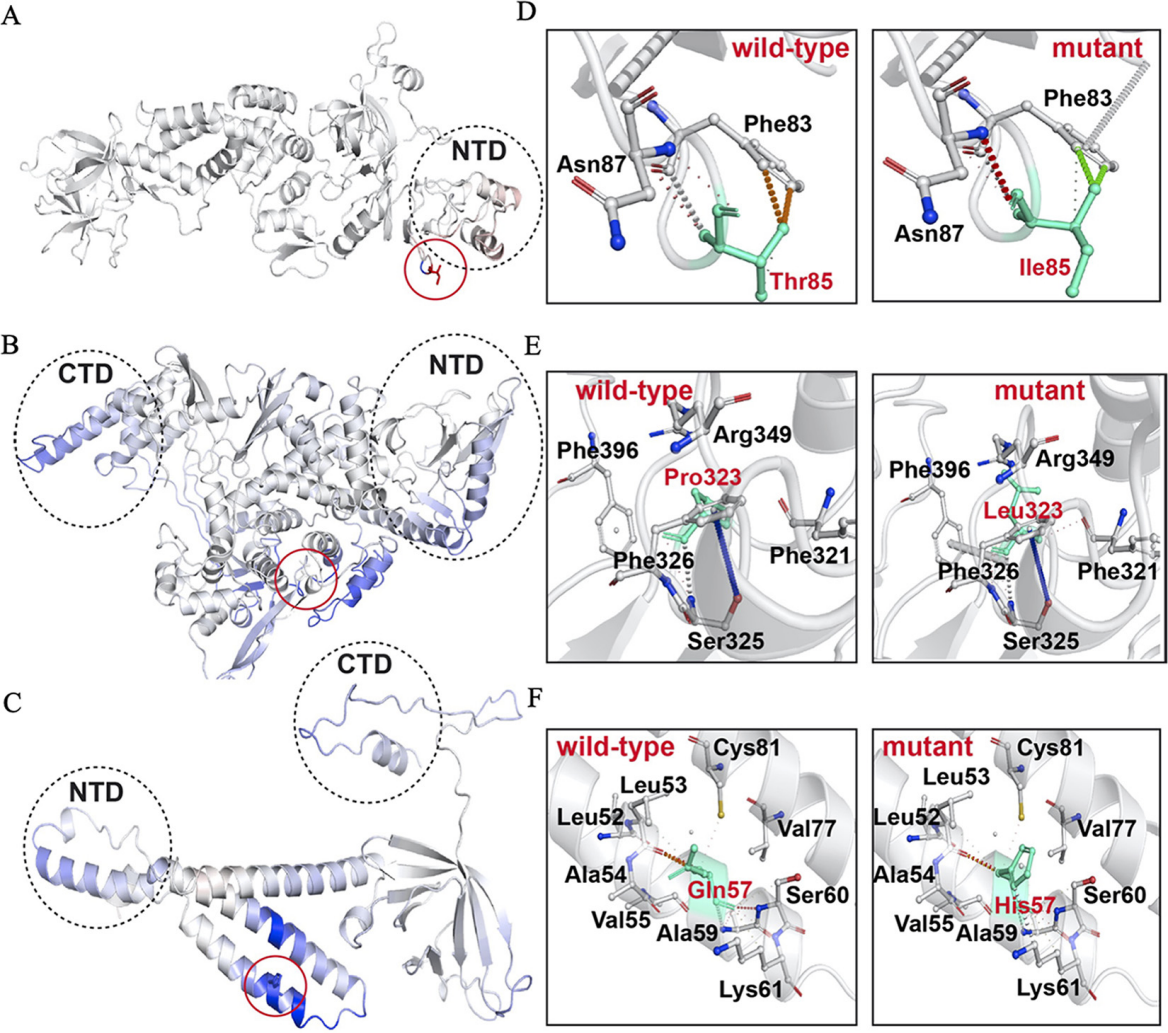
Visual representation of mutant protein dynamicity and intramolecular interactions of wild-type and mutant residues with proximal amino acids. Mutations are shown in stick representation and are circled in red. Red and blue indicate flexibility and rigidity, respectively. The wild type and the mutant residues are shown in cyan. Mutant residues are in red, while interacting residues are in black. Interactions are illustrated in different colors; for further interpretation of interactions, see the web version of the Arpeggio web server. (A to C) Visual representation of nsp2, RdRp, and ORF3a, respectively. (D to F) Intramolecular interactions of wild-type and mutant residues in nsp2, RdRp, and ORF3a, respectively.

Since the T85I (1059C>T) mutation was widespread among nsp2 proteins, we sought to investigate the role of this mutation in the function of this protein. The full-length 3.2-Å crystal structure of nsp2 (PDB ID 7SMW) was solved by combining cryo-electron microscopy (cryo-EM) and the recently developed AI tool AlphaFold2 (23). In the structure, there is a highly conserved zinc binding site, which indicates the role of nsp2 in RNA binding (Fig. 7A). We also studied the T85I mutation in nsp2, in which a polar threonine residue is replaced with a hydrophobic isoleucine. The PredictSNP tool revealed that this mutation is deleterious, with around a 70% confidence score. The ENCoM-based negative vibrational entropy energy (ΔΔ*S*_vib_) value suggests that this mutation confers some degree of flexibility on nsp2. It can be seen that two helices (1:19 to 28 amino acids [aa] and 2:35 to 45 aa) at the N terminus gain slight flexibility (Fig. 8A, red). Among the six predictors, four predicted a negative free energy change (ΔΔ*G*), thereby implying the destabilization of nsp2 (Table 5).

**TABLE 5:** Predicted results for the effects of mutation on functionality, stability, and flexibility of respective proteins using PredictSNP, DynaMut, and SAAFEC-SEQ web servers

Protein (mutation)	Confidence score (%) and nature of mutation (PredictSNP)	ΔΔ*S*_vib_ (kcal mol^−1^ K^−1^) and flexibility (ENCoM)	ΔΔ*G* (kcal mol^−1^)^*a*^					
			DynaMut	ENCoM	mCSM	SDM	DUET	SAAFEC-SEQ
nsp2 (T85I)	72 (deleterious)	0.021 (increase)	−0.264	−0.017	−0.125	0.460	0.151	−0.93
RdRp (P323L)	83 (neutral)	−0.225 (decrease)	0.732	0.180	−0.261	1.570	0.457	−0.77
ORF3a (Q57H)	76 (deleterious)	−0.117 (decrease)	0.597	0.094	−0.843	0.060	−0.652	−1.03

a A negative value indicates a predicted destabilizing effect, while a positive value indicates a stabilizing one.

Our results on nsp2 protein stability and flexibility are in accordance with already published reports (15). In the wild-type and mutant proteins, two identical residues (Phe83 and Asn87) interact with the wild-type and mutant residues. In both the structures, van der Waals clashes were observed between the side chain oxygen of Thr85 and aromatic carbons of Phe83 in the wild type and between the side chain methyl group carbon atom of Ile85 and aromatic carbons of Phe83 in the mutant. In the wild type, Thr85 amide group oxygen and nitrogen interact with surrounding amide group atoms of Phe83 and Asn87 through hydrophobic, van der Waals, and polar interactions. However, in the mutant protein, similar interactions were noted, but a polar-van der Waals clash was observed between Asn87 and Ile85. This might be the cause of the predicted instability of the T85I mutation in nsp2. The wild-type and mutant interactions are illustrated in Fig. 8D.

### Mutations in nsp3.

The nsp3 protein in coronavirus has been shown to antagonize the innate immune responses (24). The mutations in the nsp3 macrodomain region lead to enhanced type I interferon (IFN) responses and reduced viral replication (25). Understanding the dynamics of mutations in nsp3 might provide clues to SARS-CoV-2 evasion of type I IFN signaling. We identified a synonymous mutation, 3037C>T (F106F), that was positively correlated with 241C>T in the 5′ UTR, 23403A>G (D614G) in the spike, and 14408C>T (P323L) in the RdRp of SARS-CoV-2. Though silent, 3037C>T (F106F) was shown to disrupt the mir-197-5p target sequence (26). mir-197-5p was shown to be associated with some other viruses also (27–29), indicating its role in viral biology. The 14408C>T (P323L) mutation was shown to increase the mutation rate among SARS-CoV-2 isolates, whereas 23403A>G (D614G) has been shown to contribute to the infectivity of the virus (16). The co-occurrence of all these mutations in >87% of the genomes further points to their critical role in driving viral evolution.

### Mutation in RdRp.

RdRp (nsp12) of SARS-CoV-2 is important for viral replication and transcription. This protein is also believed to be the most prominent target for potential antiviral drugs (30). Therefore, understanding the mutations in this protein is critical for RdRp-based drug designs. The mutation 14408C>T (P323L) in RdRp was present in >89% of genomes, suggesting that this mutation is now a part of the circulating genomes. Apart from its widespread presence, this mutation was correlated with some other mutations, including 241C>T in the 5′ UTR, 3037C>T in nsp3, and 23403A>G in the spike, with high correlation coefficients of 0.73, 0.94, and 0.94, respectively. Interestingly, 14408C>T and 23403A>G mutations were reported in patients with severe COVID-19, in contrast to those with mild infections, suggesting their possible role in disease severity (31). Owing to the widespread presence of P323L mutation in RdRp, we sought to study its effect on the stability of the wild-type protein.

The 2.83-Å crystal structure of RdRp in complex with nsp7, nsp8, nsp9, and helicase was determined using cryo-electron microscopy (32). The RdRp structure has the RdRp domain (367 to 920 aa) (33) and N terminus (60 to 249 aa), which adopts the nidovirus RdRp-associated nucleotidyltransferase (NiRAN) structural scaffold (34). Another region (4 to 118 aa) is composed of two helices and five β-strands that are antiparallel. Additionally, the short β-strand (215 to 218 aa) was observed in RdRp, which is highly ordered in SARS-CoV-2 compared to SARS-CoV. This β-strand has contact with other β-strand residues (96 to 100 aa) and thus increases the conformational stability of RdRp in SARS-CoV-2 (33).

The P323L mutation is present on RdRp interface domain, especially in the loop region, which connects the interface domain’s three helices to the same domain’s three β-strands (Fig. 7B). An earlier study suggested that this mutation enhances the processivity of RdRp (35). It is predicted to be functionally neutral, with a notable confidence score of 83%. This mutation results in a conformationally rigid proline ring being replaced by a flexible side chain containing a leucine residue. Though the wild-type and mutant residues are hydrophobic, their conformational flexibility must be the deciding factor for protein stability and flexibility. Nonetheless, this mutation significantly rigidifies RdRp (Fig. 8B), and ΔΔ*S*_vib_ was also observed to be much lower (Table 5). Results show that this mutation has a strong communication network in RdRp and impacts various helices and β-sheets. The P323L mutation is located in a loop formed by the β-strand (328 to 335 aa) and helix (304 to 320 aa); thus, these two secondary structures gain rigidity. However, a helix-proximal mutation gained greater rigidity than other regions of RdRp. The helices at the N- and C-terminal domains also gained rigidity due to this mutation. All-atom simulation data also suggested that the P323L mutation reduces the flexibility of RdRp, which is in line with our results (36). Three ΔΔ*G* value predictors predicted stabilization and the remaining three predicted destabilization, but the ΔΔ*G* stabilization values are considerably higher than the destabilization values. Mohammad et al. performed 200-ns all-atom molecular dynamics simulation by calculating free energy (Δ*G*) of the wild-type and mutant RdRp and confirmed that P323L increases the stability of RdRp (36). Hence, this mutation stabilizes the RdRp structure. Analysis of the RdRp wild-type and mutant interaction revealed that there are more interactions in the mutant than the wild type. The wild-type and mutant residues are surrounded by Phe321, Ser325, Phe326, Arg349, and Phe396 residues. In the wild type, only a single polar interaction between Ser325 and Pro323 residues is observed, while in the mutant, two additional hydrogen bonds with Ser325 and Phe326 and polar interaction with Phe349 were observed. Thus, it can be considered that higher stability in the mutant comes from these interactions. Figure 8E shows the interactions in the wild-type and mutant RdRp.

### Mutations in the ORF3a protein.

ORF3a is the largest accessory protein of SARS-CoV-2 and plays a key role in the viral infection cycle. Moreover, this protein is essential for viral replication, and mutations in this protein are associated with higher mortality rates (37). The mutation 25563G>T (Q57H) in the SARS-CoV-2 ORF3a gene has been shown to be associated with decreased death and increased cases of COVID-19 (38). We further observed that 25563G>T (Q57H) mutation is positively correlated with the 1059C>T mutation in nsp2, whereas it is negatively correlated with 28881G>A, 28882G>A, and 28883G>C mutations in the nucleocapsid gene. Our observations are in agreement with previous studies which identified similar associations within the genomes of SARS-CoV-2 isolated in Israel (14). The ORF3a functional domains are vital for SARS-CoV-2 infectivity, virulence, ion channel synthesis, and the release of the virus (39). A recent study showed that ORF3a in SARS-CoV-2 has a weaker potential for proapoptotic activity than SARS-CoA ORF3a, which might be linked to the infectivity of the viruses (40). Furthermore, another study confirmed that ORF3a binds to the homotypic fusion and protein sorting (HOPS) complex and prevents autolysosome formation (41). ORF3a is also considered a potential vaccine and drug target (42, 43).

The experimental structure of ORF3a protein (PDB ID 6XDC) was determined using cryo-EM at 2.1-Å resolution. ORF3a protein has three main regions: the N terminus (1 to 39 aa), the cytoplasmic loop (175 to 180 aa), and the C terminus (239 to 275 aa) (44). Figure 7C shows the structure of ORF3a protein. In the Q57H mutation, a glutamine polar residue is replaced by a polar and basic histidine residue. This mutation is situated in the helix region of ORF3a protein and is predicted to be deleterious, having a 76% confidence score (Table 5). Similar observations were reported in earlier studies (39, 45). The ΔΔ*S*_vib_ value implied that the ORF3a protein gains rigidity and becomes less flexible due to the appearance of this mutation. Similar to RdRp, the mutant residue in ORF3a protein also has a wide communication dynamics. This single mutation in the helix increases the rigidity of the whole ORF3a protein (Fig. 8C). Our findings are in agreement with a previous study on the impact of the Q57H mutation in the protein (15).

Based upon the ΔΔ*G* values, this mutation was predicted to be destabilizing (Table 5). The wild-type and the mutant residues are in close proximity to Leu52, Leu53, Ala54, Val55, Ala59, Ser60, Lys61, Val77, and Cys81. In the wild-type protein, two hydrogen bonds are observed in Ser60 and Lys61 amide bond amino groups with the amide carbonyl group of the wild-type residue. These identical hydrogen bonds are also present in the mutant structure. Other types of interactions, such as polar and hydrophobic, were observed in the wild-type and the mutant ORF3a protein. However, there are two new clashes seen in the mutant structure between the histidine ring and Lys61 and mutant amide group and Leu53 residue. Thus, the overall number of clashes has increased in the mutant, and this might be the factor responsible for the destabilization of ORF3a protein under the influence of the Q57H mutation. This mutation was predicted to have significant potential to alter the ORF3a conformation and lead to disruption of intramolecular hydrogen bonds in ORF3a (38). Our findings that Q57H causes destabilization of ORF3a are in agreement with the previous study. The wild-type and the mutant interactions are illustrated in Fig. 8F.

### Mutation in the spike protein.

Spike protein is a homotrimer protein that studs the surface of SARS-CoV-2, giving it a crown-like shape. The spike protein of SARS-CoV-2 consists of two subunits that are covalently attached to each other. One of the subunits, S1, binds to the ACE2 receptor on the target cells, whereas the S2 subunit helps anchor the spike protein to the cell membrane (46, 47). The D614G mutation in the spike protein has been shown to increase the infectivity of the virus. In our previous study (48), we characterized the effect of D614G mutation on protein activity and suggested that the mutation led to decreased protein stability but enhanced protein movement. In this study, we observed a correlation of the 23403A>G (D614G) mutation in the spike with the mutations 241C>T (5′ UTR), 3037C>T (nsp3), and 14408C>T (RdRp). The presence of these mutations in >96% of the genomes suggests their critical role in viral pathogenesis. The above-mentioned mutations had replaced the wild-type sequences by June-July 2020 (Fig. 6A to D). Therefore, a better understanding of these comutations via further experimentation is urgently required.

### Mutations in the nucleocapsid protein.

Nucleocapsid protein is one of the most conserved proteins among SARS coronaviruses (49). This protein is known to interact with viral RNA as well as the viral membrane protein to aid virion assembly. This protein is also shown to play a role in regulating host immune responses (50) and cellular apoptosis (51). The nucleocapsid protein of SARS-CoV-2 acts as a viral RNA interference (RNAi) suppressor and has been shown to antagonize cellular RNAi pathways (52). Thus, understanding the role of mutations in modulating the function of this protein becomes important. The mutations 28881G>A, 28882G>A, and 28883G>C in the nucleocapsid are positively correlated with each other. Of these, the mutations at 28881 and 28883 are missense mutations, whereas the mutation at 28882 is synonymous. Since the mutations in nucleocapsid protein are known to increase the infectivity and virulence of the virus (53), the correlation of these mutations with other mutations warrants further study. Interestingly we observed that these three mutations in the nucleocapsid were negatively correlated with two other mutations, 1059C>T in nsp2 and 25563C>T in ORF3a. It was further observed that a very small number of haplotypes (0.06 to 0.17%) had both mutations in the same genome (Table 2). The absence of these mutations from the same genome implies their possible negative impact on viral evolution and pathogenesis. In our recent study (48), we probed the impacts of these two mutations, 28881 (R203K) and 28883 (G204R), on the nucleocapsid protein structure, function, and dynamics. Though we performed the analysis of each of these mutations separately, a recent study investigated the synergistic effect of these comutations in the nucleocapsid protein function (54). It was observed that these comutations increase the fitness, infectivity, replication, and virulence of SARS-CoV-2. These comutations were shown to increase the phosphorylation of the viral nucleocapsid protein and to confer enhanced resistance to glycogen synthase kinase-3 (GSK-3), thereby leading to efficient viral replication.

### Impact of mutations on protein dynamics.

The protein structure and functions are significantly altered by the insertion of single-point mutations (55–57). Investigating the structural or functional impacts of point mutations in all proteins can be achieved using a suite of computational tools, including NMA models (58), Gaussian network models (GNM) (59), anisotropic network models (ANM) (60), elastic network models (ENM) (61), discrete molecular dynamics (DMD) (62), all-atom molecular dynamics (AAMD) simulation (63), and protein evolutionary data. Therefore, we employed these tools to probe the effects of mutations on protein structures. The predicted results of the mutations are given in Table 5.

### Linear mutual information analysis of the mutants.

**FIG 9:**
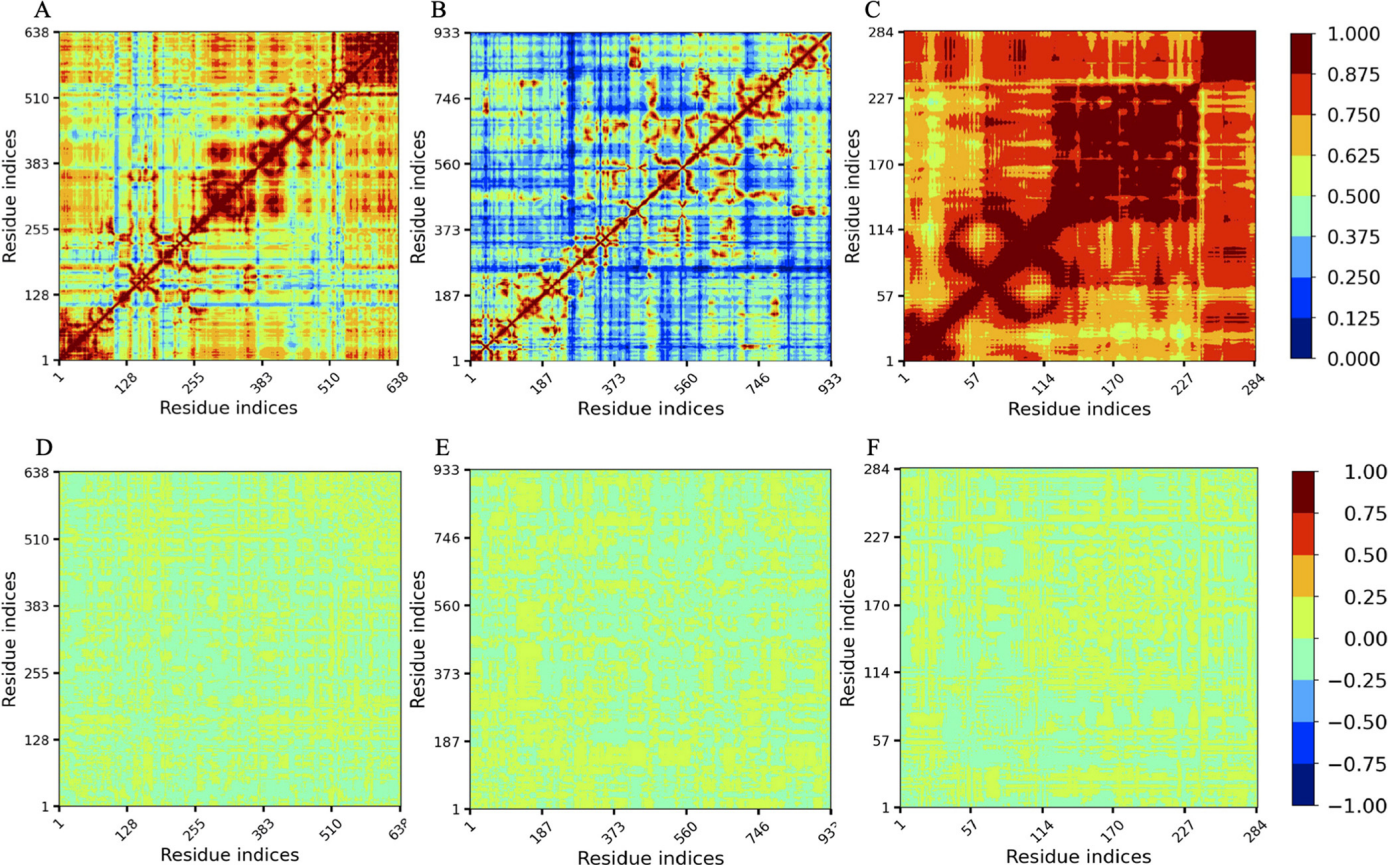
nLMI correlation plots. (A to C) Wild-type nsp2 (A), RdRp (B), and ORF3a (C). (D to F) Correlation difference plots of wild-type and mutant nsp2 (D), RdRp (E), and ORF3a (F). The degree of correlation is indicated by the color.

The normalized linear mutual information (nLMI) gives insight into the protein residue network and dynamic correlation. Figure 9 illustrates the nLMI correlation and correlation difference plots of nsp2, RdRp, and ORF3a proteins along with their mutants. It can be observed that the nsp2 and ORF3a protein residues are strongly correlated (>0.625) compared to RdRp, where correlation among residues is considerably lower (<0.500) (Fig. 9A to C). However, to understand the impacts of every single mutation in each protein, we obtained correlation differences between the wild-type and mutant structures of all the three proteins. In the correlation difference plots, the yellow regions indicate no or a very slight correlation (0.00 to 0.25), whereas cyan regions indicate slightly negative anticorrelation between the residues (Fig. 9D to F). In RdRp and ORF3a mutant structures, residues have significantly less correlation. However, the nsp2 mutant structure’s residues are slightly anticorrelated. Thus, the T85I mutation in nsp2 causes a slight disruption in the structure that can be considered destabilization of the nsp2 mutant structure. However, P323L in RdRp and Q57H in ORF3a do not result in notable destabilization of the mutant structures.

### Conclusion.

Since the onset of the SARS-CoV-2 pandemic in December 2019, the virus has significantly mutated. The mutations in the virus have led to the emergence of mutants that have the capacity to dodge vaccine and antiviral therapies. Therefore, understanding the dynamics of mutations in the viral genome is of utmost importance. To this end, we performed a time series analysis of viral sequences to understand the origin and frequency of significant mutations present in the SARS-CoV-2 circulating genomes. The meta-analysis approach was used to identify significant mutations in the SARS-CoV-2 sequences. Pearson correlation and hierarchical clustering were then used to identify the correlations and the clusters among the significant mutations. We identified 16 comutations that had an absolute Pearson correlation coefficient of >0.4 and were present in >30% of the genomes analyzed in this study. We identified a strong correlation coefficient (>0.73) for the mutations 241C>T in the 5′ UTR with 3037C>T (F106F) in nsp3, 23403A>G (D614G) in spike, and 14408C>T (P323L) in RdRp. The co-occurrence of these mutations was found in >86% of the genomes that were studied, suggesting that these comutations were part of the same haplotypes. The area plot analysis revealed that these mutations replaced the respective wild-type sequences by June-July 2020. In this study, we were able to capture negative correlations of the mutations 28881G>A, 28882G>A, and 28883G >C in the nucleocapsid gene with mutations, including 1059C>T in nsp2 and 25563G>T in ORF3a, implying that a haplotype will not harbor 28881G>A, 28882G>A, and 28883G>C nucleocapsid mutations along with 1059C>T in nsp2 or 25563G>T in ORF3a. However, the combined effect of these mutations having negative correlations in the viral replication still needs to be investigated.

To investigate the impacts of T85I, P323L, and Q57H mutations in nsp2, RdRp, and ORF3a proteins, respectively, on their structural stability and flexibility, we employed structure and sequence-based tools. ΔΔ*G* and ΔΔ*S*_vib_ were used to evaluate the stability and flexibility of proteins, respectively. From the free energy calculation, T85I and Q57H mutations in nsp2 and ORF3a proteins, respectively, disrupt the residual network in the wild-type protein and destabilize the wild-type protein, while P323L in RdRp brings stability to the wild-type protein by adding new contacts between the residues. Also, consensus predictors were used to predict the impacts of mutation on protein functions. It was noted that T85I in nsp2 and Q57H in ORF3a were found to be deleterious, which implies that they alter the protein functions. However, P323L in RdRp was predicted to be neutral, which suggests that this mutation does not have any impact on protein function. The graph theory-based nLMI correlation was also obtained for the wild-type and mutant structures of three proteins to understand the residue communication in proteins. The nsp2 and ORF3a residues have a greater correlation than with RdRp. The correlation difference plot suggests that compared to nsp2, a significant correlation was observed in RdRp and ORF3a under the influence of the mutations. Thus, it can be observed that the latter two mutations increase the residue correlation in RdRp and ORF3a proteins, while in the case of nsp2, there was no significant correlation difference observed, indicating a slight or no impact of mutation on the structure of nsp2. The fact that some of the mutations have destabilizing effects but have very high frequency suggests that these might play some role in viral fitness. Further experimentation is required to study the effect of these comutations on viral transmission and pathogenesis.

## MATERIALS AND METHODS

### SARS-CoV-2 genomic sequences.

Since the onset of the SARS-CoV-2 pandemic in 2019, the virus is continuously evolving thereby resulting in the emergence of several variants. The availability of SARS-CoV-2 genomic sequences has been instrumental in understanding viral evolution and pathogenesis. To gain an in-depth understanding of the mutational landscape of SARS-CoV-2, we sought to analyze SARS-CoV-2 genomic sequences in a time series manner. All the SARS-CoV-2 genomic sequences were collected in a monthwise manner (based on the sample collection month) from the Virus Pathogen Resource (ViPR) database (64). The database was accessed on 18 April 2021, and only complete genomic sequences of SARS-CoV-2 were downloaded for further processing. In order to obtain high-quality genomic sequences, only complete genomes, which were around 15% of the total sequences, were used in this study. Additionally, an in-house Python script was written to check for the presence of unusual bases in the sequences included in this study. In the current study, SARS-CoV-2 sequences were collected from January 2020 to March 2021, resulting in 59,541 complete genome sequences.

### Meta-analysis of SARS-CoV-2 genomic sequences.

Once the SARS-CoV-2 genomic sequences were obtained and categorized by collection month, we performed a meta-analysis on these sequences to identify significant mutations among them. The genomes from various months were analyzed with respect to the genomes collected in the month of January 2020. The genomes obtained during the initial phase of infections tend to be close to the wild type, with few mutations, compared to the genomes collected at the later stages of the infection. For the identification of significant mutations, a metadata-driven comparative analysis tool (META-CATS) was used (65). All the analyses were performed with default settings, and mutations with *P* values of >0.05 were considered significant. We obtained a considerable number of significant mutations for each month. Notably, SARS-CoV-2 genomic sequences collected in December 2020 did not yield any significant mutations. Therefore, sequences collected in December 2020 were not included for further analysis.

### Pearson correlation coefficient.

In order to identify the correlation among significant mutations in the SARS-CoV-2 circulating genomes, Pearson correlation was used. Pearson correlation measures the linear correlation between two variables. An empty matrix of 55,759 by 29,903 was created using the NumPy module of Python. In this matrix, the number of rows represents the number of SARS-CoV-2 genomic sequences that were analyzed, and the number of columns represents the length of the reference SARS-CoV-2 genome. In this matrix, the occurrence of mutation at a particular position was represented by the number 1, whereas nonoccurrence of the mutation at a specific position was represented by the number 0. All comparisons were made with the SARS-CoV-2 reference genome, GenBank accession no. NC_045512.2. We used the “corr” method of the pandas library (66) to implement Pearson correlation.

### Hierarchical clustering.

To validate the results obtained from the Pearson correlation, the hierarchical clustering technique was applied; this method groups similar objects together. Since highly frequent mutations tend to play a critical role in the evolution of the virus (17), the 25 most significant mutations that were present in more than 10% of the genomes were tested for similarity using the hierarchical clustering technique. The figure_factory method from the plotly library of Python was used to perform the hierarchical clustering on the binary matrix of the 25 most significant mutations. The pdist and squareform methods from the SciPy library of Python (67) were used to create the dendrogram with a heat map. The dendrogram, together with the heat map, represents the significant mutations that are clustered. All these analyses were performed in Python version 3.8.5.

### Protein structure and model preparation.

Once the comutations in SARS-CoV-2 were identified, several computational tools were used to investigate the effect of the mutations (that constitute the comutations) on the respective protein structure. Of these mutations, 241C>T in 5′ UTR does not get translated into an amino acid. The mutations 3037C>T (F016F in nsp3) and 28882G>A (R203R in nucleocapsid protein) are synonymous and hence were not included for further analysis. A detailed analysis of three other mutations, including 23403A>G (D614G in spike protein), 28881G>A (R203K in nucleocapsid protein), and 28883G>C (G204R in nucleocapsid protein) was performed in our recent study (48) and thus not explored in this study. Therefore, in this study, we targeted three mutations—1059C>T (T85I in nsp2), 14408C>T (P323L in RdRp), and 25563G>T (Q57H in ORF3a protein)—to probe their impact on the protein structure and function. The crystal structures of these proteins were obtained from the Protein Data Bank (PDB). Analysis of these protein structures revealed that they had some missing amino acids. Hence, we employed a deep learning-based protein modeling tool, RoseTTAFold (68), to add missing residues to these proteins. The nsp2 protein (PDB ID 7MSW) is 638 amino acids long, and in the crystal structure, the first three residues at the N terminus were missing. RdRp (PDB ID 7CYQ) is 942 amino acids long, and 1 to 3 amino acids at the N terminus and 930 to 942 amino acids at the C terminus are missing in the structure. The ORF3a protein (PDB ID 6XDC) is only 284 amino acids long, but a large number of residues from both termini (1 to 39 aa at the N terminus and 239 to 284 aa at the C terminus) and six residues (175 to 180 aa) are missing in the protein structure. RoseTTAFold modeled all these missing residues in these proteins except for the nine histidine residues at the C terminus of RdRp. These three modeled proteins were further analyzed for mutation effects.

### Functional impact of mutations.

To investigate the effect of mutation on protein function, we used the widely popular PredictSNP web server (69). This web tool is composed of a suite of six different predictors, including predictor of human deleterious single nucleotide polymorphism (PhD-SNP), multivariate analysis of protein polymorphism (MAPP), screening of nonacceptable polymorphism (SNAP), polymorphism phenotyping v1 (PolyPhen-1), polymorphism phenotyping v2 (PolyPhen-2) and sorting intolerant from tolerant (SIFT) to predict whether a given mutation is deleterious or neutral. PredictSNP gives a consensus prediction score using these six predictors. These six predictors make use of different methods to predict the nature of the mutation. PhD-SNP, MAPP, SNAP, PolyPhen-1, SIFT, and PolyPhen-2 utilize a support vector machine, physicochemical characteristics and a protein sequence alignment score, a neural network approach, an expert set of empirical rules, a protein sequence alignment score, and naive Bayes, respectively (69), to predict whether a given mutation is deleterious or neutral. To calculate the PredictSNP score, the following equation is employed:

(1) $$\text{PredictSNPScore}=\;\frac{\sum_{i=1}^{N}\left({\text{δ}}_{i}S_{i}\right)}{\sum_{i=1}^{N}S_{i}}$$

where δ*_i_* is an inclusive prediction (−1, neutral; +1, deleterious), *S_i_* indicates the transformed confidence scores, and *N* is the number of predictors. The PredictSNP consensus score is between −1 and +1, where −1 to 0 corresponds to a neutral and 0 to +1 to a deleterious mutation.

### Effect of mutations on protein dynamics.

The normal mode analysis (NMA)-based DynaMut (70) web tool was utilized to probe the effects of a single mutation in each protein on its stability and flexibility. The folding free energy change (ΔΔ*G*) was calculated to exactly predict the stability of the protein under the influence of the mutations. In addition to its own ΔΔ*G* prediction, DynaMut also predicts ΔΔ*G* using NMA-based ENCoM (Elastic network contact model) (71) and other structure-based predictors, like the mutation cutoff scanning matrix (mCSM) (72), site-directed mutator (SDM) (73), and DUET (74). The free energy change indicates the stability of the proteins by measuring the energy difference between the wild-type and mutant proteins. Additionally, DynaMut employs ENCoM to predict vibrational entropy energy (ΔΔ*S*_vib_). The values of ΔΔ*S*_vib_ are calculated for wild-type and mutant proteins by screening their all-atom pair interactions. We utilized the protein sequence-based SAAFEC-SEQ (single amino acid folding free energy changes-sequence) tool (75) to validate the DynaMut predictions for wild-type and mutant proteins. This tool utilizes different protocols, such as protein sequence properties, evolutionary details, and physicochemical properties, to calculate the ΔΔ*G* value.

### Linear mutual information.

To understand the nature of dynamics and fluctuations in the protein structures, dynamical cross-correlation (DCC)- and LMI-based approaches were employed in this study (76–79). Since DCC cannot calculate the correlation of atoms moving concurrently in perpendicular directions (80), we applied normalized LMI (nLMI) to overcome this limitation. To calculate the nLMI of wild-type and mutant proteins, we employed the Python-based correlation-plus 0.2.1 tool (80), which uses PDB files as the input. During the calculation, the program uses the anisotropic network model (ANM) to generate 100 models of wild-type and mutant proteins, and the correlation is thus obtained using these models. Additionally, we calculated the difference in correlation between wild-type and mutant proteins. To calculate nLMI between residues *i* and *j*, the following equation was used;

(2) $${\text{LMI}}_{\mathrm{ij}}=\frac{1}{2}[\text{ln}\left(\text{det}C_{i}\right)+\text{ln}\left(\text{det}C_{j}\right)-\text{ln}(\text{det}C_{\mathrm{ij}})]$$

where $C_{i}=\;\langle x_{i}^{T}x_{i}\rangle$, $C_{j}=\langle x_{j}^{T}x_{j}\rangle$, and $C_{\mathrm{ij}}=\langle {\left(x_{i},x_{j}\right)}^{T}\;\left(x_{i},x_{j}\right)\rangle$; also, $x_{i}=R_{i}-\langle R_{i}\rangle$ and $x_{j}=R_{j}-\langle R_{j}\rangle$, where *R_i_* and *R_j_* are the atom *i* and *j* position vectors, det = Determinant of protein residue cross correlation metrices. In the nLMI calculation, the LMI was considered greater than or equal to 0.3 and the distance threshold was less than or equal to 7 Å. The values 0 and 1 indicate no correlation and complete correlation of residues, respectively.
