# Supplementary material for: Time Series Analysis of SARS-CoV-2 Genomes and Correlations among Highly Prevalent Mutations
Source: Microbiol Spectr. 2022 Sep 7;10(5):e01219-22. doi: 10.1128/spectrum.01219-22 (PMC9603882; doi:10.1128/spectrum.01219-22)
Supplement: Supplemental file 1 — Table S1. Download spectrum.01219-22-s0001.pdf, PDF file, 0.08 MB [file spectrum.01219-22-s0001.pdf]

Time series analysis of COVID-19 data identifies correlations among highly prevalent mutations

Neha Periwal[1], Shravan Rathod[2], Sankritya Sarma[3], Gundeep Singh[4], Avantika Jain[1,5], Ravi P. Barnwal[6], Kinsukh R Srivastava[7], Baljeet Kaur[8], Pooja Arora[3] and Vikas Sood[1]

Affiliations:

1. **Biochemistry Department, SCLS, Jamia Hamdard**
2. **Chemistry Department, Smt. S.M. Panchal Science College, Talod, India**
3. **Zoology Department, Hansraj College, University of Delhi**
4. **Humber College, Toronto, Canada**
5. **DIPSAR, Delhi**
6. **Biophysics Department, Punjab University**
7. **Division of Medicinal and Process Chemistry, CDRI, Lucknow**
8. **Department of Computer Science, Hansraj College, University of Delhi**

Supplementary Table 1: Table showing the number of genomes that were analysed in this study. The number of genomes collected in month wise manner is also shown in the table.

Month	Number of Genomes
January 2020	141
February 2020	289
March 2020	8453
April 2020	5023
May 2020	3250
June 2020	3379
July 2020	7359
August 2020	4833
September 2020	2225
October 2020	1528
November 2020	1997
December 2020	3782
January 2021	9633
February 2021	859
March 2021	6970
Total	59541
